# Clinical significance for diagnosis and prognosis of POP1 and its potential role in breast cancer: a comprehensive analysis based on multiple databases

**DOI:** 10.18632/aging.204255

**Published:** 2022-09-09

**Authors:** Xiao He, Ji Wang, Honghao Yu, Wenchang Lv, Yichen Wang, Qi Zhang, Zeming Liu, Yiping Wu

**Affiliations:** 1 Department of Plastic Surgery, Tongji Hospital, Tongji Medical College, Huazhong University of Science and Technology, Wuhan 430030, Hubei, China; 2 Department of Emergency, The People’s Hospital of China Three Gorges University, The First People’s Hospital of Yichang, Yichang 443000, Hubei, China

**Keywords:** breast cancer, bioinformatics, The Cancer Genome Atlas, Gene Expression Omnibus, POP1

## Abstract

Background: Breast cancer (BC) is one of the most common cancers in women. The discovery of available biomarkers is crucial for early diagnosis and improving prognosis. The effect of POP1 in BC remains unrevealed. Our study aims to explore the expression of POP1 in BC and demonstrate its clinical significance and potential molecular mechanisms.

Methods: The Cancer Genome Atlas (TCGA) BC cohort transcriptome data and corresponding clinical information were downloaded. GSE42568 cohort, GSE162228 cohort, GSE7904 cohort, and GSE161533 cohort in the Gene Expression Omnibus (GEO) database were used as verification groups. R software and several web tools were used for statistical analysis. Moreover, the proliferation, transwell, wound healing experiments, and flow cytometry were used for *in vitro* investigation.

Results: Compared with normal breast tissue, POP1 expression was up-regulated in BC tissue with a higher mutation rate. POP1 had good diagnostic value for BC and could be utilized as a new marker. POP1 was significantly correlated with multiple pathways in BC and played an important role in the immune infiltration of BC. High-POP1 expression patients were more prone to be responded to immunotherapy and had a significantly higher percentage of immunotherapy response rate. Moreover, POP1 promoted proliferation and migration and inhibited apoptosis in BC cells.

Conclusions: POP1 expression was up-regulated in BC and was associated with a poor prognosis. Patients with high-POP1 expression were more likely to be responded to immunotherapy. Our study can provide a potential marker POP1 for BC, which is beneficial in the diagnosis and treatment of BC.

## INTRODUCTION

Breast cancer (BC) seriously affects the physical and mental health of women because of its high incidence [1]. Surgery is still the most common treatment for BC, and other treatments include chemotherapy, radiotherapy, neoadjuvant therapy, targeted therapy, and immunotherapy [2]. With the popularization of monitoring methods and the development of surgical techniques, early BC patients can get a better prognosis [3]. But, there is still a significant proportion of patients with poor prognoses, such as estrogen receptor (ER)-negative [4, 5]. Although immunotherapy has achieved good outcomes in many solid tumors, its efficacy in BC is limited and only a small percentage of patients can produce a lasting response [6–8]. This may be related to the “cold tumor” feature of BC [9]. Further exploration of potential targets that may be used in BC diagnosis, prognostic monitoring, and efficacy prediction is warranted.

The POP1 gene encodes a ribonuclease that locates in the nucleus and plays a role in tRNA preprocessing [10]. The POP1 protein is also an autoantigen in patients with connective tissue disease and is involved in suppressing inflammation [11, 12]. In recent years, the potential of POP1 has been preliminarily elucidated. For example, Zhu et al. found that POP1 was a novel prognostic marker of colorectal cancer through bioinformatics analysis [13]. Liang et al. constructed a pyroptosis-related prognosis model for gastric cancer, in which POP1 was a gene in the model [14]. However, the role of POP1 in BC has not been intensively investigated.

The relationship between single cells, the microenvironment, and the immune system inside the tumor dictates the progression rate of cancer [15]. Numerous cancers have been ameliorated using immunotherapies such as chimeric antigen receptor (CAR)-T therapy and immune checkpoint blockade (ICB) [16, 17]. Specifically, in renal cell carcinoma, lung adenocarcinoma, and melanoma, antibodies that target immune activation regulators, including programmed cell death protein-1 (PD-1), cytotoxic T-lymphocyte-associated protein 4 (CTLA-4), and programmed death ligand-1 (PD-L1), have significantly increased survival [18]. In recent years, great progress has been made in the treatment of cancer patients with ICBs [19].

Numerous factors generated from cancer cells could regulate the tumor-infiltrating lymphocytes (TILs) response. Immune checkpoints shield tumor cells from immune attacks by inhibiting aberrant activation of TIL response [20]. PD-L1 is a kind of 33 kDa type 1 transmembrane protein, and it can establish a main immune checkpoint via combining with PD-1 on T cells. Therefore, cancer cells can inhibit TILs activation, expansion, and acquisition of effector functions to avoid T cell-mediated immune surveillance [21]. PD-L1 overexpression in solid tumors contributes to the CAR-T cell poor treatment because of mediating the exhaustion of CAR-T cells [22]. Thereby, in the tumor microenvironment (TME), targeting the PD-L1/PD-1 axis might potentially reinvigorate depleted CAR-T cells and TILs [23–25]. However, the response rate for ICB monotherapy is often below 40%, and many patients do not benefit from it [26]. At present, the role of POP1 in anti-tumor immunity is rarely studied. Exploring the relationship between POP1 and the tumor immune microenvironment can contribute to a stronger indicator of tumor status and prediction of treatment response and efficacy.

Bioinformatics analysis provides a convenient and in-depth analytical method to explore the genomics and proteomics of cancer [27]. Gene Expression Omnibus (GEO) database and The Cancer Genome Atlas (TCGA) database are the two most commonly used databases. Their widespread application has led to the successful identification of novel markers and predictive models or signatures for BC. Here, we explored the significance of POP1 in BC through bioinformatics analysis. We combined TCGA data and GEO data sets for comprehensive analysis and validation. Through the analysis of the expression, Kaplan Meier, immune microenvironment, and immune therapy response to explore the role of POP1 in BC. In addition, the Cell Counting Kit-8 (CCK-8) assay, wound healing experiments, transwell, and flow cytometry were applied for in-depth investigations. Our research revealed that POP1 overexpression was correlated with BC poor prognosis and could provide a breakthrough for the clinical diagnosis of BC.

## MATERIALS AND METHODS

### Expression analysis of POP1 in BC TCGA data and multiple validations in GEO data sets

Firstly, TCGA data was downloaded for analysis. Data sets GSE7904, GSE42568, GSE161533, and GSE162228 in the GEO database were used for validation (Table 1). All data were log2 transformed for subsequent analysis. Then, the POP1 expression was analyzed via the “ggplot” package in R software.

**Table 1:** Characteristics of included studies.

	**Country**	**Platform**	**Normal**	**Mean0**	**SD0**	**Tumor**	**Mean1**	**SD1**
TCGA	USA	NR	99	1.27	0.265	1072	1.84	0.61
GSE42568	Ireland	GPL570	17	3.11	0.859	104	3.61	0.674
GSE162228	China	GPL570	24	6.18	0.328	109	6.86	0.658
GSE7904	USA	GPL570	7	5.54	0.291	43	6.28	0.725
GSE161533	China	GPL570	28	4.49	0.184	28	4.74	0.379

### Mutation analysis and correlation analysis of clinical features

Mutation analysis was implemented in the cBioPortal database (http://www.cbioportal.org). Then, we selected the research cohort as “TCGA PanCancer Atlas Studies”, set the gene as “POP1”, and presented the mutation of POP1 in pan-cancer with a bar chart. Subsequently, we analyzed the association between POP1 and multiple clinical features. These analyses were performed in UALCAN database (http://ualcan.path.uab.edu). Select “TCGA” as the analysis object, “BC” as the tumor type, and “POP1” as the gene. The correlation between POP1 and different clinical features, including stage and lymph node metastatic status, was subsequently obtained.

### ROC curves of POP1 in different cohorts were established to assess the POP1 diagnostic accuracy

To evaluate the POP1 diagnostic value for BC, we established receiver operating characteristic (ROC) curves in 1 TCGA BC cohort and 4 GEO BC cohorts (GSE7904 cohort, GSE42568 cohort, GSE161533 cohort, GSE162228 cohort). ROC curves were built and the area under the curve (AUC) was counted via the “pROC” R package.

### Meta-analysis of multiple cohorts to further verify the role of POP1 in BC

In order to exclude independent cohort bias, we included the 5 cohorts mentioned above including the TCGA cohort, GSE42568 cohort, GSE162228 cohort, GSE7904 cohort, and GSE161533 cohort to perform the meta-analysis to verify the expression of POP1 in BC. If the 95% confidence interval (CI) of the standardized mean difference (SMD) after the combination was greater than 0, the expression of POP1 was higher in BC. The test criteria for heterogeneity were as follows: if I^2^ > 50%, the random effect model was adopted; If I^2^ < 50%, the fixed-effect model was adopted. Subsequently, analysis of sensitivity and specificity were performed to verify the meta-analysis results’ credibility. The accuracy of the meta-analysis was verified via establishing the summary receiver operating characteristic (SROC) curve and counting the AUC value. Egger test and Begg test were utilized to assess the publication bias size. The “meta” and “forest” packages in R software and the Stata software were applied to complete the above analysis.

### Survival analysis of POP1 to explore its prognostic value

POP1 survival analysis was conducted on the TCGA cohort. According to the median POP1 expression, BC patients were divided into high- and low-POP1 groups. The prognosis difference between two groups was analyzed by survival analysis which was performed via the “Survival” R package.

### Gene Ontology (GO) enrichment analysis and Gene Set Enrichment Analysis (GSEA)

GO Enrichment analysis was carried out to explore the correlation of molecular functions and signaling pathways between POP1 and BC. The clusterProfile R package was utilized to analyze differential genes and their functional enrichment between the high- and low-POP1 group. Subsequently, GSEA was conducted to calculate the corresponding enrichment score for each known gene set.

### Correlation analysis of POP1 and immune microenvironment

The differences in immune cell infiltration levels were investigated by the “CIBERSORT” database. The pheatmap R package was utilized to establish an immune landscape for BC. The “ggplot” R package was applied to establish a histogram of different immune cell infiltration. Subsequently, the Spearman correlation test was conducted to calculate the association between POP1 expression and distinct immune cells. Finally, the differential infiltration and high correlation of immune cell types were obtained via the Venn diagram, and the different infiltrated immune cell types were intersected with highly correlated immune cell types.

### Construction of the POP1-related nomogram

Nomograms are diffusely used in cancer prognosis, mainly because they can reduce statistical prediction models to a single numerical estimate of an event probability, which can be customized to each patient’s situation. To better assess the BC patients’ prognosis, we established a nomogram according to the expression level and clinical correlation of POP1. The “Regplot” R package was utilized for the construction of the nomogram. The patient ID was: TCGA-E2-A14Z. We then constructed the 3-, 4-, and 5-year calibration curves of the nomogram to evaluate its accuracy.

### Exploration of immune characteristics and prediction of immunotherapy response

According to the “estimate” R package (version 4.0.2), three scores were counted. The expression of 53 immune checkpoint-related genes was extracted to analyze for differential expression. We used Tumor Immune Dysfunction and Exclusion (TIDE) analysis to accurately simulate immune evasion and predict tumor immunotherapy response [28]. Patients with a TIDE score > 0 were considered unresponsive to immunotherapy, and patients with TIDE score < 0 were considered responsive to immunotherapy. In addition, a subclass mapping algorithm was performed to determine whether patients in high- and low-POP1 groups were suitable for immunotherapy [29]. Moreover, differential expression analysis of drug sensitivity (half maximal inhibitory concentration, IC50) was performed to identify suitable potential targeted drugs.

### Cell transfection and real-time reverse transcription-PCR (qRT-PCR)

MDA-MB-231 and MCF-7 cells (American Type Culture Collection, Manassas, VA, USA) were cultured in Dulbecco’s modified Eagle’s medium (DMEM; Gibco, Carlsbad, CA, USA) added with 10% fetal bovine serum (FBS; Gibco, Carlsbad, CA, USA) at 37° C with 5% CO2 atmosphere. All sequences of small interference RNA (siRNA; Ribobio, Guangzhou, China) for POP1 were available in Supplementary Table 1. The Lipofectamine 3000 Transfection Reagent (Invitrogen, Carlsbad, CA, USA) was utilized to transfect. After 24 h, qRT-PCR was utilized to assess transfection effectiveness. Total RNAs were isolated from cultivated BC cells and fresh clinical tissue samples using TRIzol (Takara, Japan). The 1st Strand cDNA Synthesis Kit (Yeasen, Shanghai, China) was utilized to generate cDNA. With SYBR GreenTM Master Mix (Yeasen, Shanghai, China), the qRT-PCR was performed in QuantStudio1 (ABI Q1, USA). All qRT-PCR primer sequences were designed and launched by Tsingke (Beijing, China) and were available in Supplementary Table 2.

### Cell viability and invasion assay

The CCK-8 method (Yeasen, Shanghai, China) was applied to assess the proliferative abilities of two cells. After seeding in 96-well plates, MDA-MB-231 and MCF-7 cells were cultured to 40% confluence at 37° C. 10 μL CCK-8 reagent was added after transfection. The optional density (OD) was detected at 450 nm at 0, 24, 48, and 72 h after CCK-8 reagent supplementation.

Cells were resuspended with 200 μL serum-free DMEM and implanted into 24 well transwell migration chambers (8 μm pore size; Corning, NY, USA) inner chambers to conduct transwell migration assays. 700 μL DMEM medium containing 20% FBS, as an attractant, was injected into the bottom chambers. The chamber membranes were soaked in 4% paraformaldehyde for 30 min after incubation for 24 h, and cotton swabs were used to wipe the upper part of the chamber to eliminate non-migrated cells. Afterward migrated cells on the membrane lower side were stained with 0.1% crystal violet for 30 min at 37° C. The ImageJ software was applied for measuring the quantity of migrated cells.

The migration abilities of siRNA transfected cells were tested by wound healing assay. Transfected MDA-MB-231 and MCF-7 cells were seeded in the 6 well plates and grown to 90% confluent cell monolayer. With the 200 μL micropipette tip, the single-cell layer was scratched in each well. Then, the detached cells and debris were washed with phosphate-buffered saline (PBS), and the serum-free DMEM medium was added to each well. The 24 h horizontal distance of migrated cells was observed and measured using microscopy and ImageJ software.

Flow cytometry was applied to detect cell apoptosis with Annexin V-FITC/PI apoptosis kit (MultiSciences, Hangzhou, China). Cells were plated into 6-well plates and cultured for 24 h with siRNAs. Cells were collected, stained via Annexin V-FITC/PI for 5 min, and detected via flow cytometry.

### Availability of data and materials

The datasets provided for this study can be found in online repositories. The name and accession number(s) of the repository/repositories can be found in the article Supplementary Materials.

## RESULTS

### POP1 expression in multiple BC cohorts

**Figure 1:**
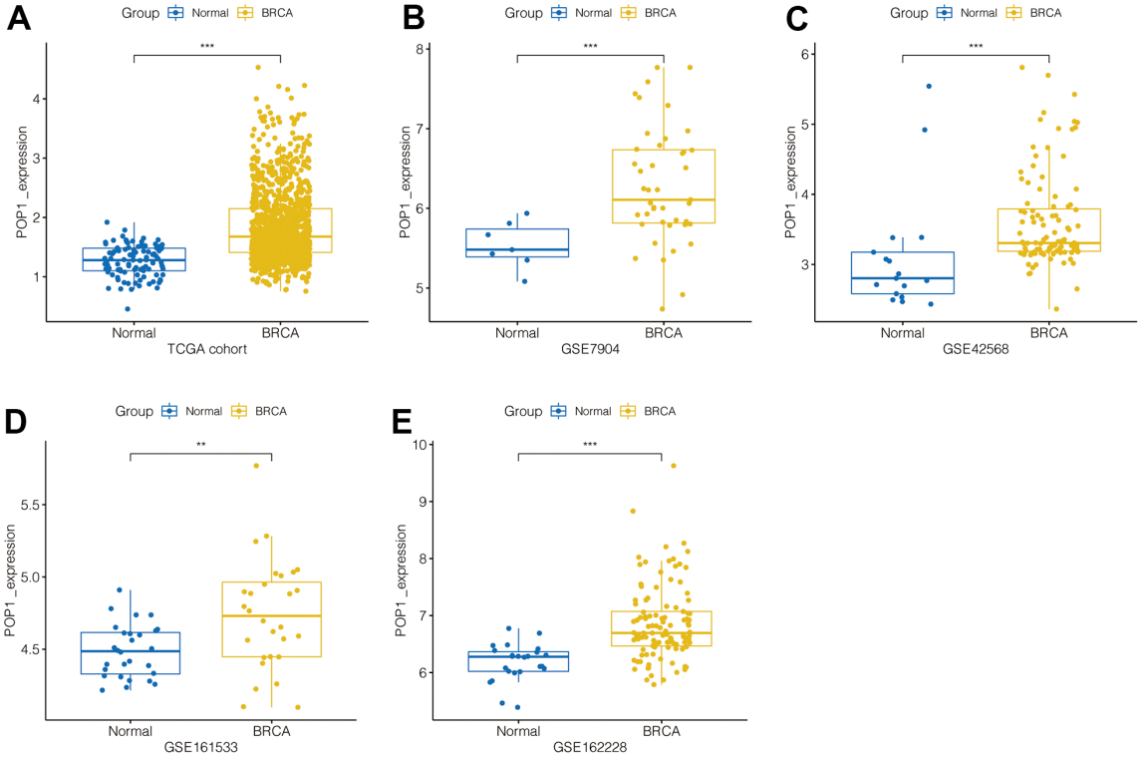
**POP1 expression in BC.** POP1 expression between non-BC tissues and BC tissues based on The Cancer Genome Atlas (TCGA) and Gene Expression Omnibus (GEO) datasets. (**A**) TCGA cohort. (**B**) GSE7904. (**C**) GSE42568. (**D**) GSE61533. (**E**) GSE162228. ** P < 0.01; *** P < 0.001. Abbreviations: TCGA, The Cancer Genome Atlas; GEO, Gene Expression Omnibus.

Firstly, in the TCGA BC cohort, POP1 expression was significantly higher in BC tissue than in normal breast tissue (Figure 1A). Subsequently, by using the GEO datasets, POP1 was overexpressed in BC tissues in the GSE7904 cohort, GSE42568 cohort, GSE161533 cohort, and GSE162228 cohort (Figure 1B–1E). Therefore, the POP1 was an up-regulated indicator in BC tissues from TCGA and GEO datasets.

### Mutation analysis and correlation analysis of clinical features

**Figure 2:**
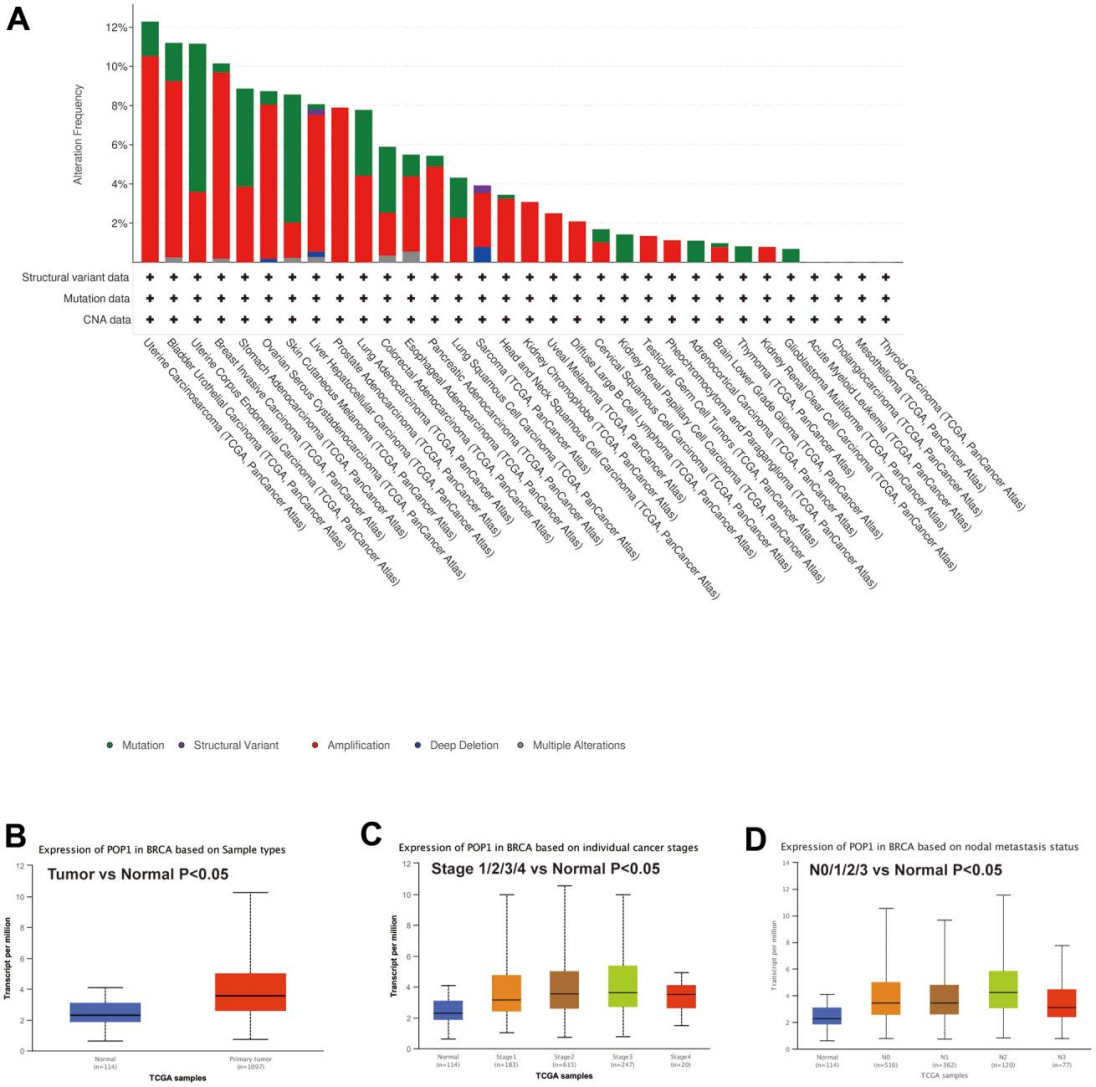
**Mutation status of POP1 gene based on cBioPortal and the relationship between POP1 expression and clinical features of BC based on UALCAN databases.** (**A**) Mutation analysis of the cBioPortal database showed that POP1 was the fourth most common mutation in BC. (**B**) UALCAN database also suggested that POP1 was more highly expressed in BC than in normal breast tissue. (**C**, **D**) Subgroup analysis verified that POP1 expression was higher in BC than in normal tissues regardless of stage or lymph node metastasis status.

First, mutation analysis of the TCGA pan-cancer cohort revealed that POP1 mutation frequency in BC ranked fourth among all tumor types (Figure 2A). Subsequent analysis of the UALCAN database also suggested that the POP1 expression was higher in BC than in normal breast tissue (Figure 2B). Subgroup analysis demonstrated that POP1 was overexpressed in BC regardless of stage or lymph node metastasis status (Figure 2C, 2D).

### ROC curves of POP1 in different cohorts were constructed to evaluate the diagnostic accuracy of POP1

**Figure 3:**
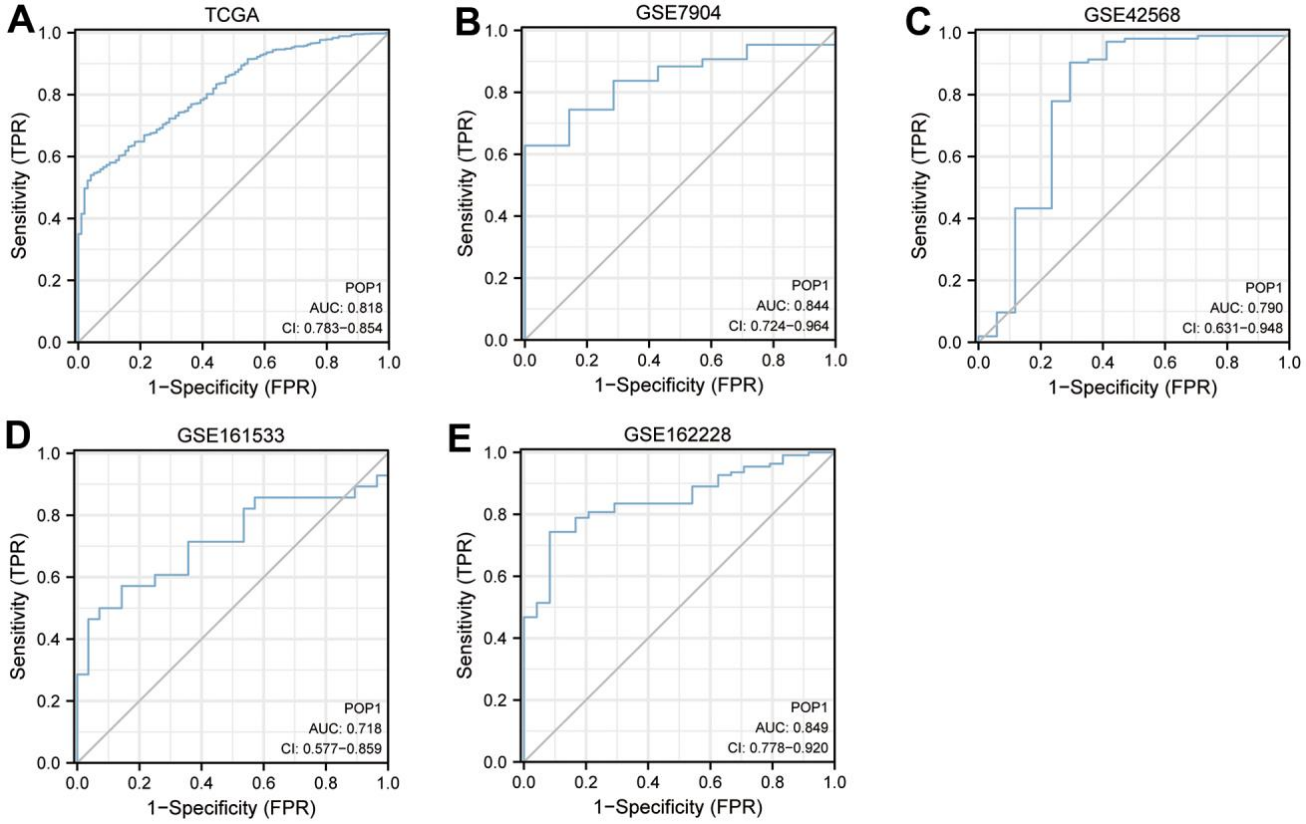
**Diagnosis value of POP1.** The receiver operating characteristic (ROC) curve to obtain the area under the curve (AUC) value of POP1 in different BC cohorts. (**A**) The Cancer Genome Atlas (TCGA) cohort. (**B**) GSE7904. (**C**) GSE42568. (**D**) GSE61533. (**E**) GSE162228. Abbreviations: ROC, Receiver operating characteristic; AUC, Area under the curve; TCGA, The Cancer Genome Atlas.

To verify the diagnostic accuracy of POP1 for BC, we constructed ROC curves in multiple cohorts and calculated the corresponding AUC values. The AUC of POP1 in the TCGA cohort was 0.818 (Figure 3A). The AUC of POP1 was 0.844 (Figure 3B), 0.790 (Figure 3C), 0.718 (Figure 3D), and 0.849 (Figure 3E) in GSE7904 cohort, GSE42568 cohort, GSE161533 cohort, and GSE162228 cohort respectively. This demonstrated that POP1 had high accuracy in the diagnosis of BC.

### Meta-analysis of multiple cohorts to demonstrate the POP1 role in BC

**Figure 4:**
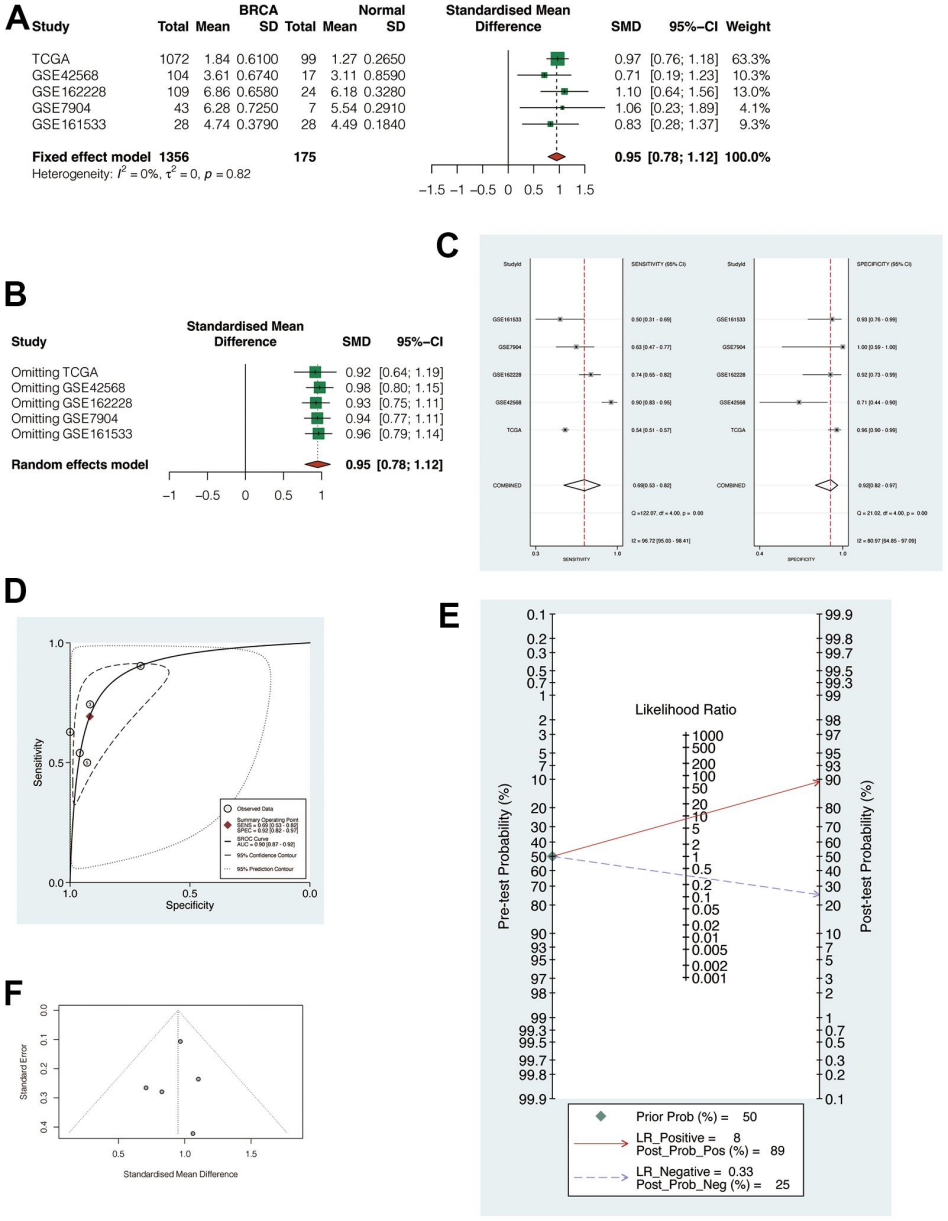
**Meta-analysis of multiple cohorts to verify the robustness of POP1.** (**A**) Meta-analysis. The heterogeneity was < 50%, so the fixed effect model was used for analysis (SMD = 0.95, 95% CI: 0.78, 1,12). (**B**) Regardless of the exclusion of any cohort, the combined standardized mean difference (SMD) was greater than 0. (**C**) Sensitivity analysis and specificity analysis. (**D**) The summary receiver operating characteristic (SROC) curve demonstrated that the results are reliable. (**E**, **F**) Begg test and Egger test did not find significant publication bias. Abbreviations: SMD, Standardized mean difference; SROC, Summary receiver operating characteristic.

The above 5 cohorts (1 TCGA cohort, 4 GEO Cohorts) were jointly analyzed to exclude bias. The results suggested that the heterogeneity was less than 50%, so the fixed effect model was applied for analysis (Figure 4A, SMD = 0.95, 95% CI: 0.78, 1.12). This indicated that POP1 was still highly expressed in the BC group after the combination. Subsequently, it found that regardless of the exclusion of any cohort, the combined SMD was greater than 0 (Figure 4B). In addition, sensitivity analysis and specificity analysis also showed the same results, indicating that the results of the meta-analysis were relatively robust (Figure 4C). SROC curve showed that the results were reliable (Figure 4D, AUC = 0.90, 95% CI: 0.87-0.92). Begg test and Egger test did not find significant publication bias (Figure 4E, 4F).

### Analysis of POP1 survival and clinical correlation in BC

**Figure 5:**
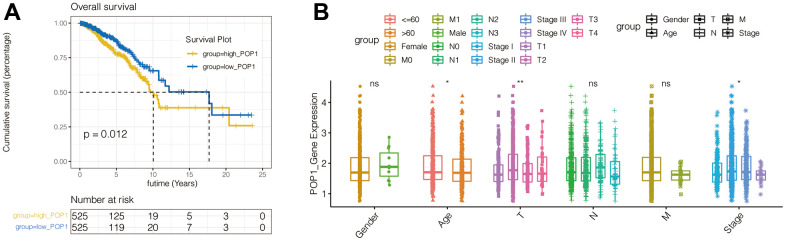
**Survival curves of overall survival (OS) and clinical characteristics analysis in BC.** (**A**) Kaplan-Meier curves of BC patients OS in patients with different POP1 expressions. (**B**) The correlation between POP1 expression and the gender, age, T stage, N stage, M stage and total stage of BC patients *P < 0.05; **P < 0.01. Abbreviations: OS, Overall survival.

According to the POP1 median expression level, BC patients were divided equally into the high- and low-POP1 group in the TCGA cohort. The results of survival analysis suggested that the high-POP1 expression was correlated with the BC poor prognosis (Figure 5A). Univariate and multivariate Cox regression analysis showed that the high expression of POP1was associated with low overall survival (OS) (Table 2, HR = 1.5, P = 0.012). This demonstrated that POP1 overexpression was independently related to low OS in BC patients. Analysis of clinical correlation suggested that POP1 expression was obviously related to age, T stage, and total stage (Figure 5B).

**Table 2:** Univariate and multivariate Cox analysis of breast cancer patients’ overall survival.

**Characteristics**	**Total(N)**	**Univariate analysis**		**Multivariate analysis**	
		**HR(95% CI)**	**P value**	**HR(95% CI)**	**P value**
T stage	1079				
T1	276	Reference			
T2	629	1.334 (0.889-2.002)	0.164	0.770 (0.337-1.757)	0.534
T3	139	1.572 (0.933-2.649)	0.089	0.793 (0.296-2.122)	0.644
T4	35	3.755 (1.957-7.205)	<0.001	1.187 (0.354-3.985)	0.782
N stage	1063				
N0	514	Reference			
N1	357	1.956 (1.329-2.879)	<0.001	1.133 (0.575-2.234)	0.718
N2	116	2.519 (1.482-4.281)	<0.001	1.771 (0.531-5.912)	0.353
N3	76	4.188 (2.316-7.574)	<0.001	3.051 (1.011-9.208)	0.048
M stage	922				
M0	902	Reference			
M1	20	4.254 (2.468-7.334)	<0.001	6.989 (1.200-40.699)	0.031
Pathologic stage	1059				
Stage I	180	Reference			
Stage II	619	1.697 (0.985-2.922)	0.057	2.266 (0.694-7.397)	0.175
Stage III	242	2.962 (1.664-5.273)	<0.001	5.012 (1.024-24.517)	0.047
Stage IV	18	11.607 (5.569-24.190)	<0.001		
Age	1082				
<=60	601	Reference			
>60	481	2.020 (1.465-2.784)	<0.001	2.393 (1.495-3.831)	<0.001
Race	993				
Asian	60	Reference			
Black or African American	180	1.525 (0.463-5.024)	0.488		
White	753	1.325 (0.420-4.186)	0.631		
Histological type	977				
Infiltrating Ductal Carcinoma	772	Reference			
Infiltrating Lobular Carcinoma	205	0.827 (0.526-1.299)	0.410		
PR status	1033				
Negative	342	Reference			
Indeterminate	4	0.826 (0.113-6.035)	0.851		
Positive	687	0.732 (0.524-1.025)	0.069		
ER status	1034				
Negative	240	Reference			
Indeterminate	2	13.088 (3.128-54.771)	<0.001		
Positive	792	0.712 (0.495-1.023)	0.066	0.467 (0.276-0.790)	0.005
HER2 status	727				
Negative	558	Reference			
Indeterminate	12	0.000 (0.000-Inf)	0.994		
Positive	157	1.593 (0.973-2.609)	0.064		
Radiation therapy	986				
No	434	Reference			
Yes	552	0.576 (0.394-0.841)	0.004	0.434 (0.267-0.704)	<0.001
POP1	1082				
Low	540	Reference			
High	542	1.538 (1.110-2.130)	0.010	1.706 (1.021-2.850)	0.041

### GSEA and GO enrichment analysis

**Figure 6:**
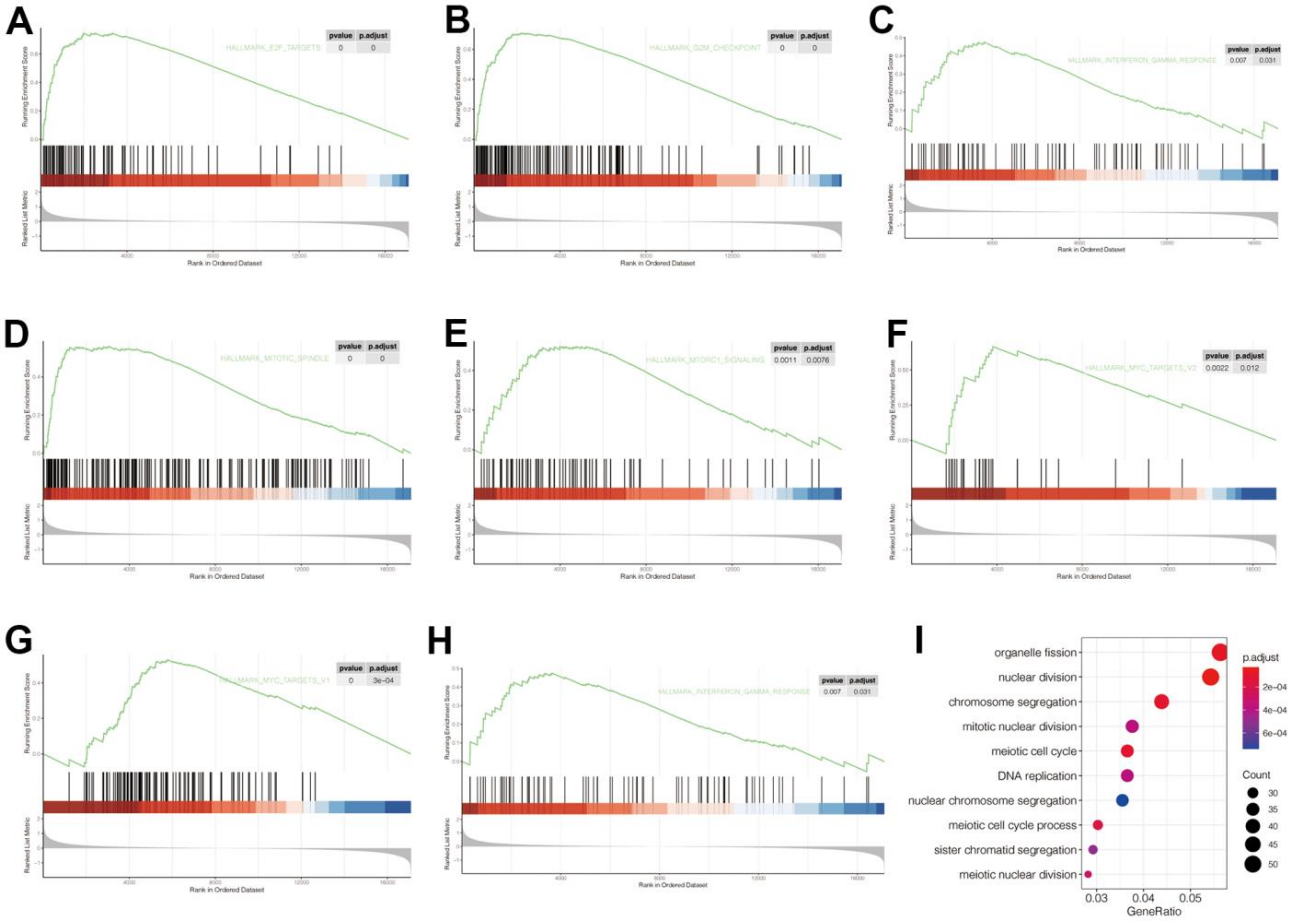
**Gene Set Enrichment Analysis (GSEA) and Gene Ontology (GO) of corresponding functions and signaling pathways associated with POP1 expression.** (**A**–**H**) The POP1 expression was significantly correlated with the E2F target, G2M checkpoint, interferon-gamma response, mitTORC1 signaling, and MYC targets. (**I**) The differentially expressed genes between the high- and low-POP1 groups were mainly enriched in organelle fission, nuclear division, and chromosome segregation. Abbreviations: GSEA, Gene set enrichment analysis; GO, Gene Ontology.

To explore the carcinogenic mechanism of POP1 in BC, it was necessary to analyze the relationship between POP1 and corresponding functions and signaling pathways. Firstly, GSEA analysis indicated that POP1 was significantly correlated with the E2F target, G2M checkpoint, interferon-gamma response, mitTORC1 signaling, and MYC targets (Figure 6A–6H). Subsequently, GO enrichment analysis verified that the differentially expressed genes were primarily interested in functional pathways, including organelle fission, nuclear division, and chromosome segregation (Figure 6I).

### Analysis of correlation between POP1 and immune microenvironment

**Figure 7:**
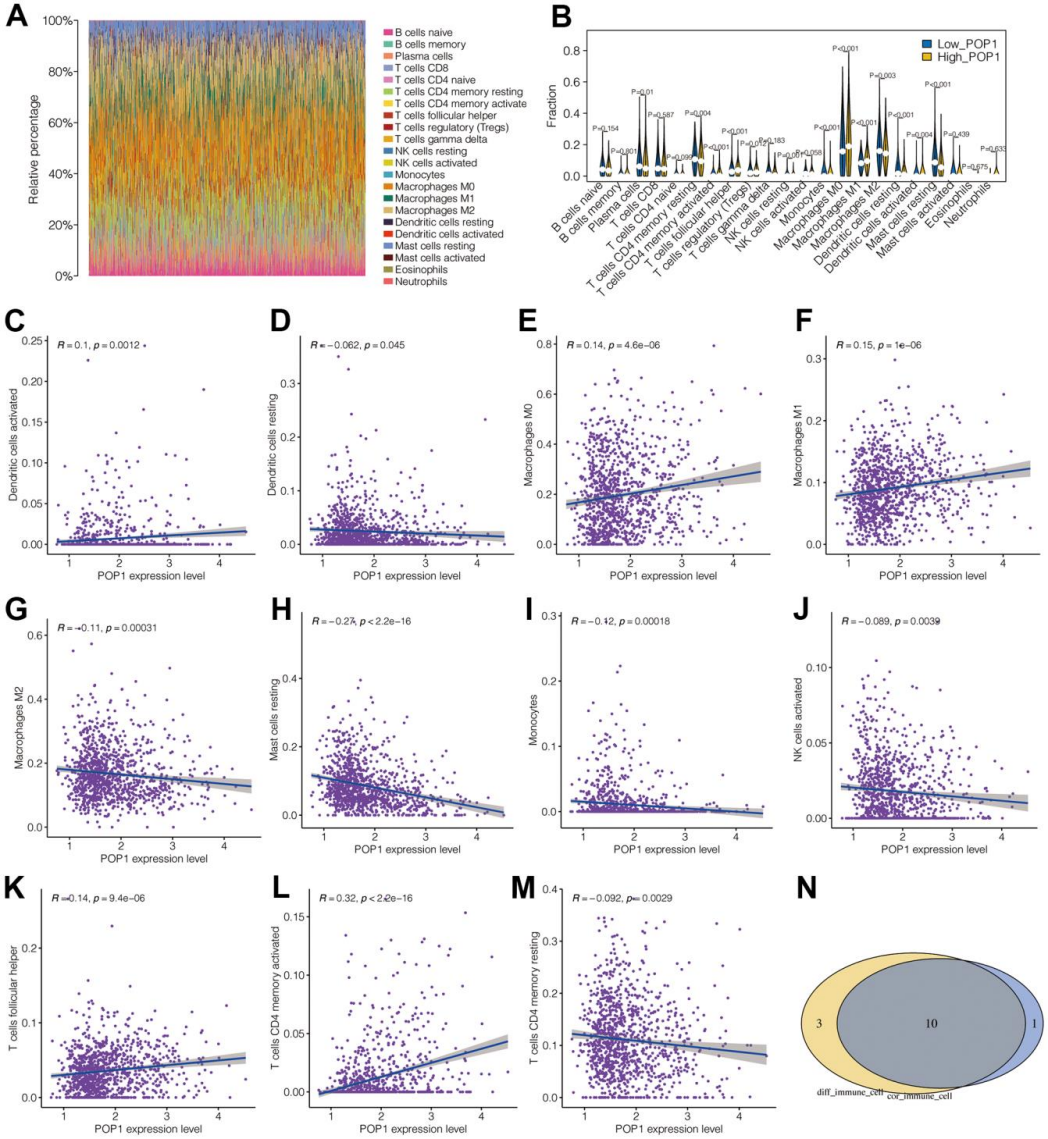
**Correlation analysis between POP1 and immune microenvironment.** (**A**) The landscape of immune cell infiltration in BC. (**B**) Difference analysis results of immune cell infiltration between the high- and low-POP1 group. (**C**–**M**) Correlation analysis showed that POP1 expression was correlated with a variety of immune cells. (**N**) A Venn diagram intersects the immune-cell types infiltrated differently with those associated with POP1.

Figure 7A showed the landscape of immune cell infiltration in BC. Through this heat map, the abundance of each immune cell was intuitively observed. Figure 7B presented different analysis results of immune cell infiltration in the two groups. T cells CD4 memory resting, T cells CD4 memory activated, Plasma cells, T cells regulatory (Tregs), T cells Follicular helper, natural killer (NK) cells resting, mast cells resting, macrophages M0, macrophages M1, macrophages M2, monocyte, dendritic cells (DCs) resting and DCs activated were differently infiltrated between the high- and low-POP1 group. Subsequent analysis of correlation verified that POP1 expression was related to many immune cells (Figure 7C–7M). Venn diagram intersected the differentially infiltrated immune cell types with highly correlated immune cell types, resulting in 10 immune cell types (Figure 7N).

### Nomogram construction based on POP1 expression

**Figure 8:**
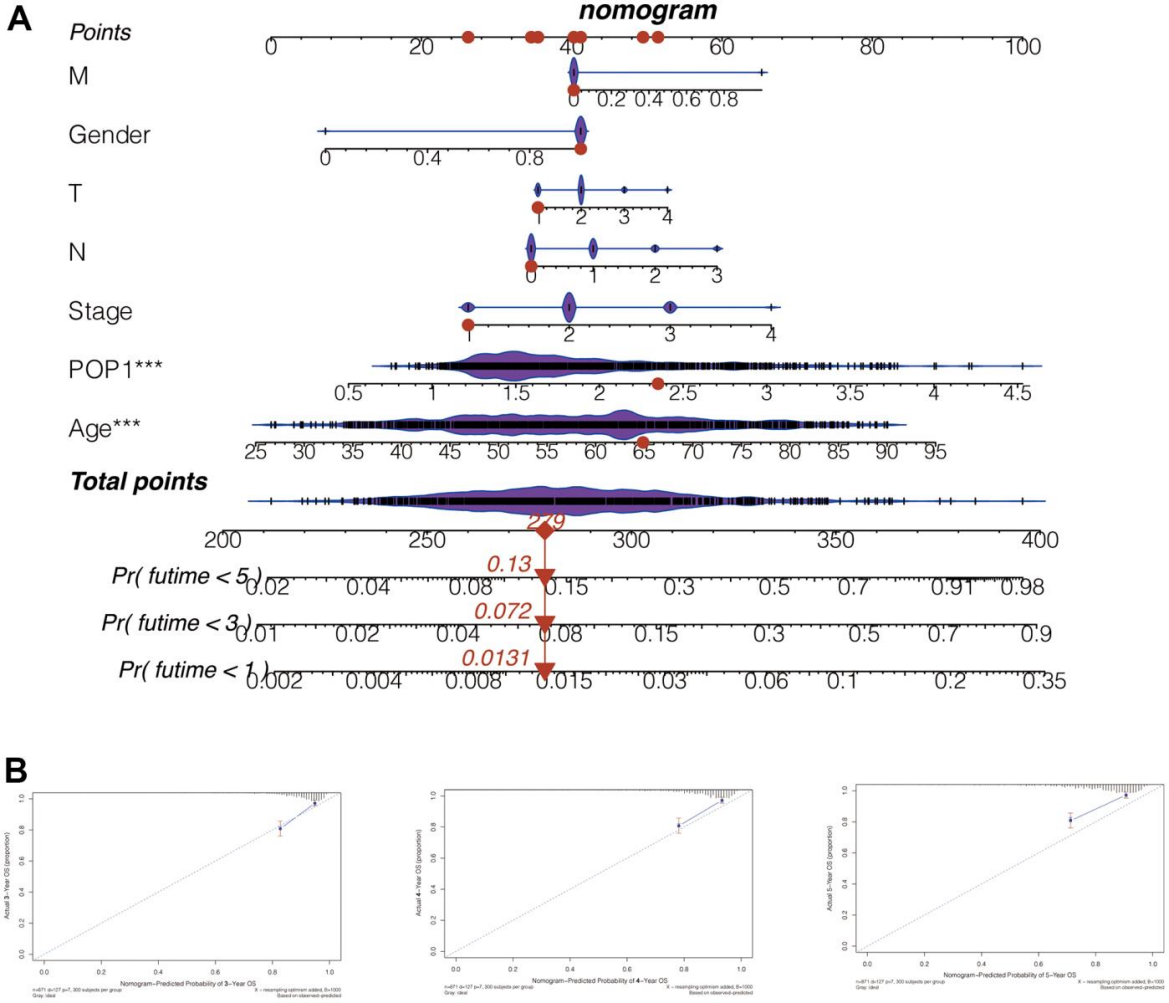
**Nomogram to evaluate patient mortality.** (**A**) The 1-, 3-, and 5-year mortality of BC patients predicted by this nomogram was 0.0131, 0.072, and 0.13, respectively. (**B**) According to the consistency between the observed and predicted values, the nomogram calibration plot of the training cohort was established. The calibration curves of 3, 4, and 5 years indicated that nomogram results are accurate.

Subsequently, we constructed a nomogram for POP1 by integrating POP1 expression and clinical data. The 1-, 3-, and 5-year mortality forecasted by this nomogram was 0.0131, 0.0720, and 0.1300, respectively (Figure 8A). Based on the agreement between observed and predicted values, a nomogram calibration diagram of the training cohort was established. The 3-, 4-, and 5-year calibration curves verified the accuracy of the nomogram results (Figure 8B).

### Analysis of immunity and immunotherapy response

**Figure 9:**
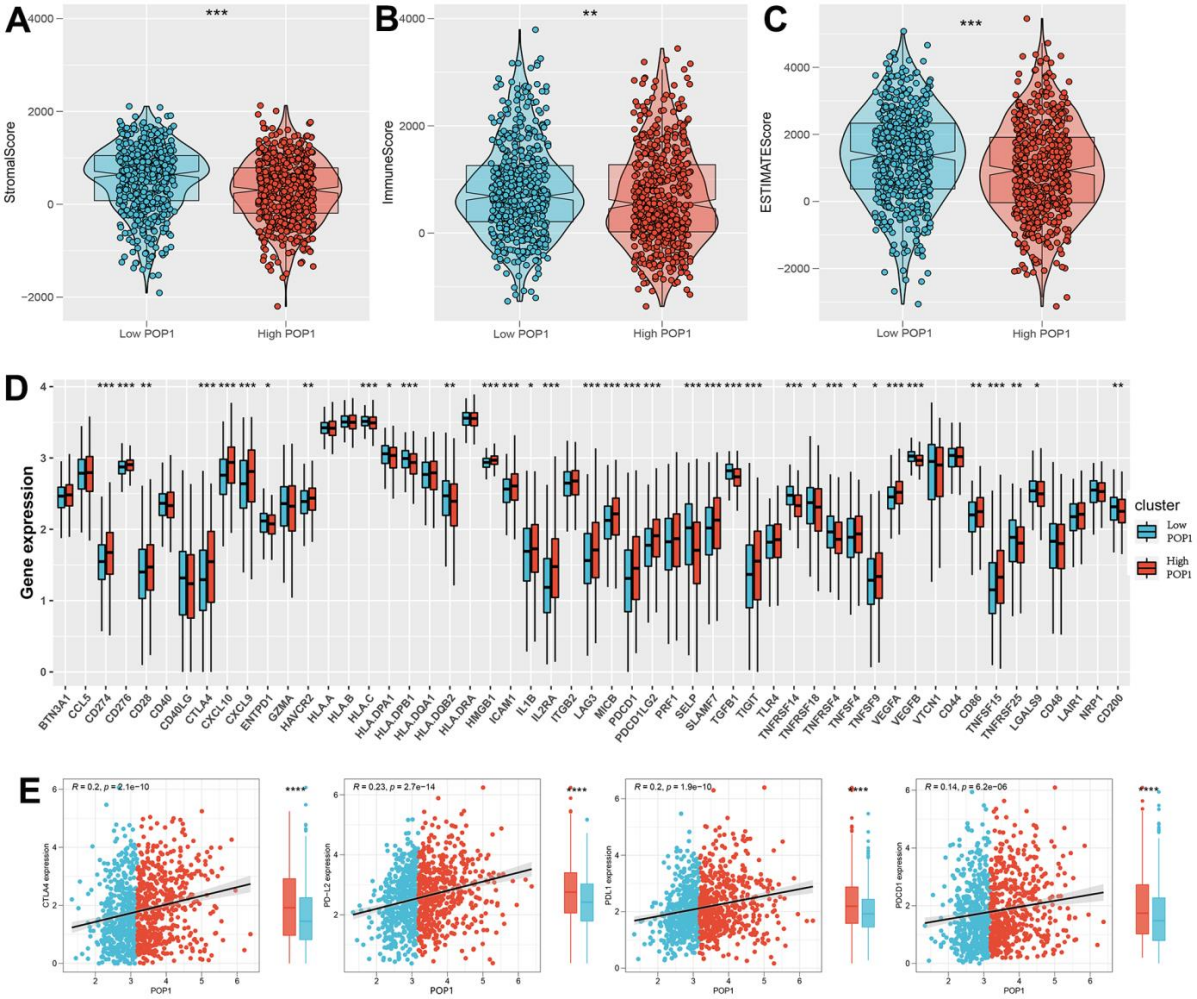
**Investigate the differences in immune characteristics between the high- and low-POP1 groups.** (**A**–**C**) Discrepancies in three scores between the high- and low-POP1 groups. (**D**) Expression differences of 53 immune characteristics between the high- and low-POP1 groups of BC patients. (**E**) Correlation analysis of four immune checkpoints in high- and low-POP1 population of BC patients. *P < 0.05; **P < 0.01; ***P < 0.001.

Calculate 3 scores for all samples of BC according to the ESTIMATE algorithm. All three scores were found to differ significantly between the high- and low-POP1 groups (Figure 9A–9C). Considering the vital role of ICB therapy in the tumor, 53 checkpoint-relevant genes were screened for differential expression analysis in the samples. The expression of most immune checkpoints differed significantly in the two groups (Figure 9D). Meanwhile, the correlation between POP1 and the four most common immune checkpoints suggested that POP1 expression was positively related to the expressions of CTLA-4, PD-1, PD-L1, and PD-L2 (Figure 9E).

**Figure 10:**
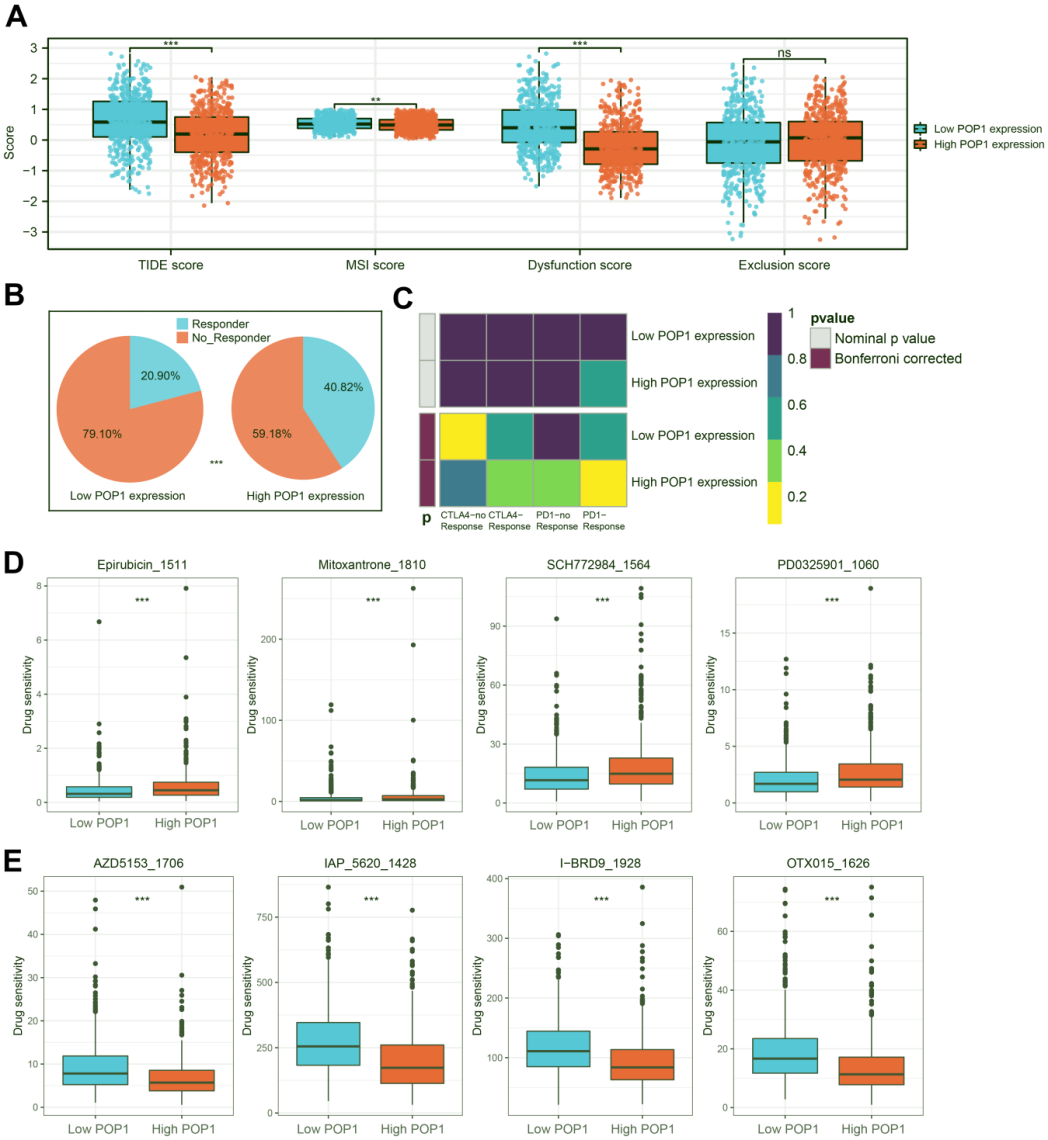
**Immunotherapy response prediction and potential drug screening.** (**A**) Distribution of Tumor Immune Dysfunction and Exclusion (TIDE) score, microsatellite instability (MSI) score, Dysfunction score, and Exclusion score. (**B**) Responder rates differed between the high- and low-POP1 groups. (**C**) A subclass mapping algorithm was used to verify the immunotherapy prediction results. (**D**, **E**) The drugs with the most significant difference in sensitivity between the high- and low-POP1 groups. **P < 0.01; ***P < 0.001. Abbreviations: TIDE, Tumor Immune Dysfunction, and Exclusion; MSI, Microsatellite instability.

Based on the TIDE algorithm, we calculated the TIDE score, microsatellite instability (MSI) score, dysfunction score, and exclusion score of all samples. The TIDE score was lower in the high-POP1 group, suggesting that immunotherapy might be more effective in the high-POP1 group (Figure 10A). Notably, the patients with the higher POP1 expression had a higher infiltration of immune characteristics. About 40.82% of high-POP1 group patients were effective for immunotherapy, while only 20.90% of low-POP1 group patients were effective for immunotherapy (Figure 10B). In addition, we performed a subclass mapping analysis to validate the immunotherapy prediction results. Consistent with these results, patients with high-POP1 expression might be sensitive to PD-1 therapy, while patients with low-POP1 expression might not be sensitive to CTLA-4 therapy (Figure 10C). Finally, IC50 estimates were performed for each sample of 179 drugs in the GDSC database by the R “oncoPredict” package and identified drugs with significant sensitivity differences in the two groups. Figure 10D, 10E suggested 4 drugs with the most obvious difference in sensitivity between high- and low-POP1 groups.

### POP1 could facilitate BC cells proliferation and migration, and inhibit apoptosis

**Figure 11:**
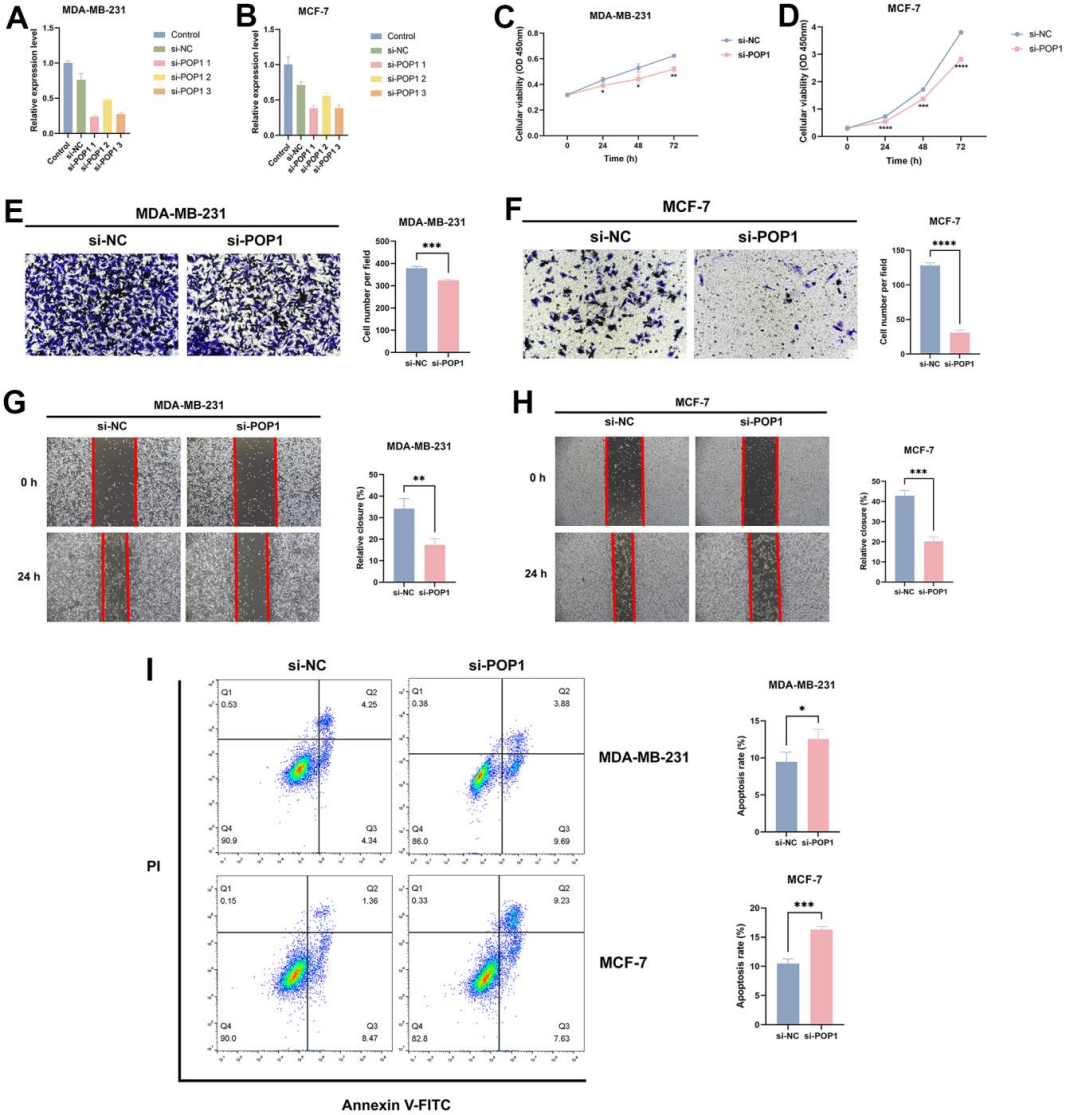
**The effect of POP1 on proliferation, migration, and apoptosis of BC cells.** (**A**, **B**) The qRT-PCR results suggested that siRNAs could successfully knock down the expression of POP1 in MDA-MB-231 and MCF-7. (**C**, **D**) Compared with the si-NC group, POP1 deficiency significantly inhibited the proliferation of MDA-MB-231 and MCF-7. (**E**–**H**) The depletion of POP1 significantly reduced the migration ability of MDA-MB-231 and MCF-7 cells, as demonstrated by transwell migration assays (magnification, 200×) and wound healing assays (magnification, 20×). (**I**) Cell apoptosis was detected via flow cytometry. *P < 0.05; **P < 0.01; ***P < 0.001; ****P < 0.0001.

For an in-depth investigation of the POP1 function *in vitro*, we detected the role of the POP1 gene in the viability and invasion of two cells. Firstly, siRNAs could significantly knock down POP1 expression (Figure 11A, 11B). CCK-8 analysis demonstrated that compared with the si-NC group, the knockdown of POP1 suppressed the proliferation of both cell types (Figure 11C, 11D). Then, the POP1 depletion reduced the invasion of both cell types (Figure 11E–11H). Finally, flow cytometry data demonstrated that POP1 remarkably suppressed BC apoptosis (Figure 11I).

## DISCUSSION

Currently, screening and early monitoring techniques have enabled many BC patients to be diagnosed at an early stage and timely intervention [30]. However, in less developed areas, especially in some developing countries, the early management of BC is not ideal [31]. The recurrence, metastasis, and drug resistance of triple-negative breast cancer (TNBC) remain major therapeutic challenges [32]. There is an urgent need to explore the tumor immune microenvironment of BC to identify novel markers to guide the diagnosis and treatment.

In this study, we revealed POP1 as a novel BC marker through an in-depth analysis and explored its role in the immune microenvironment. Firstly, the GEO and TCGA datasets were utilized to explore the POP1 expression pattern and clinical significance in BC. The combined SMD reached 0.95, indicating that POP1 was significantly overexpressed in BC. Mutation analysis showed that POP1 mutation frequency in BC was the fourth among all tumor types. POP1 had a high mutation rate in BC regardless of stage or lymph node metastasis status. Meta-analysis verified the robustness of POP1 as a prognostic marker in BC. Through ROC curve analysis, it was observed that POP1 had good performance in distinguishing BC tissues from non-BC tissues. The same conclusion was reached by the SROC analysis. Moreover, the expression of high-POP1 was independently linked to poor OS in BC patients. Meanwhile, POP1 appeared to be associated with the E2F target, G2M checkpoint, interferon-gamma response, mitTORC1 signaling, and MYC targets. The differentially expressed POP1 was primarily focused on organelle fission, nuclear division, chromosome segregation, and other functional pathways.

Furthermore, we analyzed the immune cells and correlative immune pathways in two risk groups. Patients with high-risk scores got a higher level of most immune signatures. CTLA-4, PD-1, PD-L1, and PD-L2 levels were higher in high-risk patients. PD-L1 expression has been demonstrated as a potential target for cancer immunotherapy [33]. Meanwhile, PD-1 expression increases neuronal killing of tumor cells and is linked to prolonged survival [34]. TIDE analysis revealed that the immunotherapy response rate in the high-risk group was higher than that in the low-risk group, which might be related to the level of immune-checkpoint-relevant genes. The results of subclass mapping algorithm analysis verify our findings that PD-1 checkpoint therapy was more likely to be effective in the high-POP1 group. Finally, by verifying the effect of POP1 *in vitro*, it was confirmed that POP1 promoted the viability and invasion of BC cells. These results were consistent with the previous results.

Malignant tumors often lack accurate prognostic markers. In the past, some biomarkers, such as PD-1, LAG-3, EGFR, and CD44, are found to have prognostic or therapeutic value in BC [35]. However, BC is a heterogeneous group, and these existing biomarkers are not sufficient to meet the needs [36]. It is urgent to explore new and robust targets to guide diagnosis and treatment. Our study found that POP1 expression was up-regulated in multiple BC cohorts and was linked to poor prognosis. All of these indicated that POP1 was a robust prognostic marker of BC.

GO enrichment analysis verified that the POP1 overexpression in BC was closely related to the activation of cell proliferation-related pathways. Cell cycle disorder is one of the characteristics of cancer, which can result in unbounded cell proliferation and cancer development [37, 38]. The disorder of cell proliferation and metastasis is an important reason for the occurrence and development of tumors [39]. Overall, the GSEA analysis demonstrated the preceding speculation that the POP1 overexpression appeared to be more likely associated with the enhancement of BC cell growth and metastasis. In addition, POP1 might play an important role by regulating crucial pathways, including organelle fission, nuclear division, and chromosome segregation pathways.

The BC immune microenvironment is quite complicated and heterogeneous [40]. The TME consists of the extracellular matrix, fibroblasts, cancer cells, immune cells, cytokines, chemokines, etc. However, immune cells and stromal cells, represent the primary non-tumor components of TME [41, 42]. Studies on TME verify that Effector T cell activation and the decrease in DC infiltration are two primary methods to decrease the immune response to cancer therapy [43, 44]. However, the factors that regulate the TME components and affect the immune response to treatment remain to be investigated. Thus, it is essential to understand the correlation between the prognosis and TME for exploring effective immunotherapies. The relationship between TME and immunotherapy has attracted increasing attention lately. The response to immunotherapy depends on the number of immune cells infiltrating the tumor [45, 46]. Therefore, after identifying the prognostic value of POP1 in BC, it is necessary to analyze its role in the immune microenvironment of BC. Our study not only provides an immune landscape for BC but also identifies immune cells highly associated with POP1, which has a certain reference value for immunotherapy of BC.

Immunotherapy is a promising cancer treatment strategy with significant survival benefits for some BC patients. Tumor ICBs have been proven to be an effective treatment for many malignancies. However, BC is a highly heterogeneous tumor that renders immunotherapy ineffective for many patients [47]. ICBs are approved for the therapy of particular cancer types and have been involved in miscellaneous clinical trials, however, only a few patients have favorable responses [48]. ICB antibodies targeting PD-1 or PD-L1 have the potential to treat metastatic BC, and the identification of BC patients who are responsive to immunotherapy is significant. Pembrolizumab, an anti-PD-1 antibody, has been found to enhance progression-free survival and OS in combination with chemotherapy for metastatic TNBC expressing PD-L1 [49]. However, in PD-L1-negative tumors, hormone-sensitive tumors, and pretreated advanced illnesses, the anti-PD-1/PD-L1 drug therapeutic is less noteworthy.

CTLA-4 is a surrogate target for immune checkpoint inhibition. Anti-CTLA-4 antibodies, including ipilimumab and tremelimumab, can improve the prognosis of metastatic melanoma and boost anti-tumor responses in BC [50]. After T-cell antigen exposure and activation, CTLA-4 is rapidly up-regulated, while PD-1 is up-regulated and persists during chronic T-cell stimulation. Anti-CTLA-4 and anti-PD-1/PD-L1 could react differently in distinct disease subtypes and clinical settings because of differences in these mechanistic. The latest research has emphasized novel combined methods that integrate anti-CTLA-4 with other ICBs or traditional treatments to greatly enhance the efficacy of immunotherapy in many cancer types [51].

According to 5 datasets from the TCGA and GEO databases, the POP1 overexpression in BC and its hopeful diagnostic and prognostic value were identified. Furthermore, we first explored the association between POP1 expression, immune properties, and immunotherapy response in BC patients. However, the limitations of this study still need to be addressed. We lack a BC cohort from our center to verify the prognostic value of POP1, needing to improve in the future. Moreover, our study is restricted to *in vitro* studies. In the future, more *in vitro*, especially *in vivo* studies are still needed to demonstrate the conclusions and investigate the concrete mechanism of POP1 in BC occurrence and development.

## CONCLUSIONS

Overall, POP1 is a hopeful diagnostic and prognostic target for BC. POP1 is positively related to the expression of the four most common immune checkpoints: CTLA-4, PD-1, PD-L1, and PD-L2. Patients with high-POP1 expression may be sensitive to PD-1 therapy, while patients with low-POP1 expression may not be sensitive to CTLA-4 therapy.

## Supplementary Material

Supplementary Tables
