# Supplementary material for: Clinical significance for diagnosis and prognosis of POP1 and its potential role in breast cancer: a comprehensive analysis based on multiple databases
Source: Aging (Albany NY). 2022 Sep 9;14(17):6936–56. doi: 10.18632/aging.204255 (PMC9512506; doi:10.18632/aging.204255)
Supplement: Supplementary Tables [file aging-14-204255-s001.pdf]

SUPPLEMENTARY TABLES

Supplementary Table 1. siRNA sequences for POP1.

Gene	Sense 5'-3'	Antisense 5'-3'
si-NC	CAGAAGAATGGTACAAATCCAAG	CTTCGTTCAGTATGTTAATCGT
si-POP1-1	GCAGTGCATTCTCAGTATA	TATACTGAGAATGCACTGC
si-POP1-2	GCTCGAAGATGTCACATGA	TCATGTGACATCTTCGAGC
si-POP1-3	GCAGGAAGCTCTGACTCTA	TAGAGTCAGAGCTTCCTGC

Supplementary Table 2. Primers for qRT-PCR.

mRNA	Species	Forward	Reverse
POP1	Human	AGAGGTGTAAAGCACCACAGT	GCTGTCGTGAAGTTCCAGG
GAPDH	Human	GGAGCGAGATCCCTCCAAAAT	GGCTGTTGTCATACTTCTCATGG
